# Synergistic effect of interleukin 1 alpha on nontypeable *Haemophilus influenzae*-induced up-regulation of human beta-defensin 2 in middle ear epithelial cells

**DOI:** 10.1186/1471-2334-6-12

**Published:** 2006-01-24

**Authors:** Sung-Kyun Moon, Haa-Yung Lee, Huiqi Pan, Tamotsu Takeshita, Raekil Park, Kiweon Cha, Ali Andalibi, David J Lim

**Affiliations:** 1 The Gonda Department of Cell and Molecular Biology, House Ear Institute, Los Angeles, CA, USA; 2 Department of Otolaryngology, Keck School of Medicine, University of Southern California, Los Angeles, CA, USA; 3 Department of Cell and Neurobiology, Keck School of Medicine, University of Southern California, Los Angeles, CA, USA; 4 Department of Otolaryngology, Ajou University School of Medicine, Suwon, Korea; 5 Department of Otorhinolaryngology, Hamamatsu University School of Medicine, Hamamatsu, Japan; 6 Department of Microbiology, Vestibulocochlear Research Center, Wonkwang University, Iksan, Korea

## Abstract

**Background:**

We recently showed that beta-defensins have antimicrobial activity against nontypeable Haemophilus influenzae (NTHi) and that interleukin 1 alpha (IL-1 alpha) up-regulates the transcription of beta-defensin 2 (DEFB4 according to new nomenclature of the Human Genome Organization) in human middle ear epithelial cells via a Src-dependent Raf-MEK1/2-ERK signaling pathway. Based on these observations, we investigated if human middle ear epithelial cells could release IL-1 alpha upon exposure to a lysate of NTHi and if this cytokine could have a synergistic effect on beta-defensin 2 up-regulation by the bacterial components.

**Methods:**

The studies described herein were carried out using epithelial cell lines as well as a murine model of acute otitis media (OM). Human cytokine macroarray analysis was performed to detect the released cytokines in response to NTHi exposure. Real time quantitative PCR was done to compare the induction of IL-1 alpha or beta-defensin 2 mRNAs and to identify the signaling pathways involved. Direct activation of the beta-defensin 2 promoter was monitored using a beta-defensin 2 promoter-Luciferase construct. An IL-1 alpha blocking antibody was used to demonstrate the direct involvement of this cytokine on DEFB4 induction.

**Results:**

Middle ear epithelial cells released IL-1 alpha when stimulated by NTHi components and this cytokine acted in an autocrine/paracrine synergistic manner with NTHi to up-regulate beta-defensin 2. This synergistic effect of IL-1 alpha on NTHi-induced beta-defensin 2 up-regulation appeared to be mediated by the p38 MAP kinase pathway.

**Conclusion:**

We demonstrate that IL-1 alpha is secreted by middle ear epithelial cells upon exposure to NTHi components and that it can synergistically act with certain of these molecules to up-regulate beta-defensin 2 via the p38 MAP kinase pathway.

## Background

Following the common cold, otitis media (OM), or inflammation of the middle ear, is the most frequent illness resulting in visits to physicians and the most common cause of hearing impairment in children [1]. The majority of the cases of OM are caused by three pathogens: *Streptococcus pneumoniae*, nontypeable *Haemophilus influenzae *(NTHi) and *Moraxella catarrhalis *[2,3].

Bacterial adherence to mucosal surfaces is a first step in respiratory infections. NTHi, *S. pneumoniae *and *M. catarrhalis *have all been shown to adhere to human upper respiratory epithelial cells [4-10]. Li and colleagues demonstrated that NTHi binds to and activates toll-like receptor 2 (TLR2) on the surface of epithelial cells [11]. The TLRs have been shown to be involved in the activation of many host genes, including cytokines, chemokines and antimicrobial peptides such as β-defensin 2 [12-14].

The defensins are cationic (polar) molecules with spatially separated hydrophobic and charged regions. *In vitro*, the defensins (at micromolar concentrations) have a broad spectrum of antimicrobial activity against bacteria, fungi, and even some enveloped viruses [15,16]. In humans and other vertebrates, the defensins can be divided into the α- and β-defensin subfamilies on the basis of the position and bonding of six conserved cysteine residues. The α-defensins are produced by neutrophils and intestinal Paneth's cells [17]. The β-defensins, on the other hand, are mainly produced by epithelial cells of the skin, kidneys, and trachea-bronchial lining of nearly all vertebrates [18-20]. Multiple β-defensin genes have been identified and three have been characterized at the peptide level [21-23]. β-defensin 1 is expressed constitutively by variety of cell types, while β-defensin 2 expression is highly up-regulated by exposure to inflammatory stimuli such as bacterial components or proinflammatory cytokines [24,25].

We have recently shown that both human β-defensin 1 and 2 have bactericidal /bacteriostatic activity against NTHi [26]. Moreover, in another study, we demonstrated that IL-1α can upregulate the transcription of β-defensin 2 in human middle ear epithelial cells, mediated by a Src-dependent Raf-MEK1/2-ERK signaling pathway [27]. In accord with our observations, IL-1α has also shown to be a potent activator of β-defensin 2 in intestinal epithelial cells [28]. Furthermore, the biological relevance of IL-1α as an inducer of β-defensin 2 in the tubotympanum has been demonstrated in *in vivo *studies that have shown IL-1α to be one of the cytokines induced in a rat model of OM [29].

In the present study we demonstrate that NTHi treatment of human middle ear epithelial cells results in release of IL-1α and that this cytokine and NTHi can synergistically up-regulate human β-defensin 2 (DEFB4) expression. Here, a note should be added regarding the new nomenclature of the b-defensin family of molecules . Human b-defensin 2 is now called DEFB4 and its mouse orthologue is called b-defensin 4 (Defb4). This change has created some confusion in the scientific community. Thus, to avoid confusion and remain consistent with the nomenclature used in our previous studies, will continue to refer to molecule under investigation as b-defensin 2.

## Methods

### Reagents

Recombinant interferon-γ-inducible protein-10 (IP-10), regulated upon activation, normally T-expressed, and presumably secreted (RANTES), interleukin (IL)-1α, IL-6, IL-8 and macrophage inflammatory protein-1β (MIP-1β) were purchased from Sigma (St. Louis, MO). PD98059 (ERK MAP kinase inhibitor), SB203580 (p38 MAP kinase inhibitor), and SP600125 (Jun N-terminal kinase inhibitor) were purchased from Calbiochem (San Diego, CA).

### Preparation of bacterial lysate

NTHi strain 12, originally a clinical isolate from the middle ear fluid of a child with acute otitis media, was used in this study [30]. A single colony of NTHi was harvested from a chocolate agar plate, inoculated into 30 ml of brain heart infusion broth supplemented with nicotinamide adenine dinucleotide (3.5 μg/ml) and placed in a shaking incubator overnight. The supernatant was discarded after centrifugation at 10,000 × g for 10 minutes. The pellet was resuspended in 10 ml of phosphate-buffered solution (PBS) and sonicated to lyse the bacteria. The lysate was then centrifuged at 10,000 × g for 10 min and the supernatant was collected. The protein concentration of the NTHi lysate was determined using the Bicinchoninic (BCA) protein assay and was in the range of 0.15 mg/ml.

### Cell culture

The human middle ear epithelial cell line (HMEEC), immortalized with the E6/E7 genes of human papilloma virus type 16 [31], was maintained in a 1:1 mixture of Dulbecco's modified Eagle's medium (DMEM) (Invitrogen, Carlsbad, CA) and bronchial epithelial basal medium (Clonetics, Walkersville, MD) supplemented with bovine pituitary extract (52 μg/ml), hydrocortisone (0.5 μg/ml), hEGF (0.5 ng/ml), epinephrine 0.5 (mg/ml), transferrin (10 μg/ml), insulin (5 μg/ml), triiodothyronine (6.5 ng/ml), retinoic acid (0.1 ng/ml), gentamycin (50 μg/ml), and amphotericin-B (50 ng/ml). Hela (human cervix epithelial) cells and A549 (human lung epithelial) cells were purchased from the ATCC (Manassas, VA), and maintained in DMEM containing 10% fetal bovine serum (Invitrogen), penicillin (100 units/ml), and streptomycin (0.1 mg/ml). For studying the effect of NTHi and IL-1α, the cells were seeded into six-well plates in triplicate, and were pretreated for one hour with or without chemical inhibitors of MAP kinase including PD98059, SB203580, and SP600125. All cells were maintained in a humidified atmosphere of 5% CO2 and 95% air.

### Animal studies

Eighteen, 10 week-old male C57BL/6 mice were used in this study. Three mice were for each time point including two control groups: non-treated and PBS-inoculated.

All aspects of animal handling were performed according to approved IACUC guidelines. The mice were transtympanically inoculated with 10 μl of the NTHi lysate (1:10 dilution) after anesthesia with Ketamine (5 mg/100 g). Middle ear mucosal RNA was then harvested at 6, 9, 12 and 24 hours post inoculation by irrigation of the bulla with three, 3.5 μl Trizol washes (Invitrogen). Briefly, the mice were anesthetized using Ketamine (5 mg/100 g), and then decapitated. A small incision (1 cm) was made in the retroauricular area and the cortical bone of the bulla was exposed after dissection. A hole, sized 2 mm × 2 mm, was made using the sharp scalpel followed by irrigation of the bulla with Trizol. The total Trizol volume was then increased to 200 μl and RNA was precipitated per the manufacturer's instructions. The middle ear mucosa was inflamed in all NTHi-treated mice, but effusion was not seen in all groups. No sepsis or death occurred as a result of the experimental treatment.

### Protein assay

A solid phase multiplexed protein assay in a sandwich ELISA format was performed to detect various cytokines simultaneously. Briefly, HMEEC medium was harvested 48 hours after treatment with NTHi or PBS. Pre-blotted membranes with various anti-cytokine antibodies in the BioSource Human Cytokine Set 1 Cartesian Array™ (BioSource, Camarillo, CA) were incubated with culture medium for 2 hours at room temperature after blocking nonspecific binding sites per the manufacturer's instructions. After adding a biotin-conjugated anti-cytokine antibody mixture (provided in the kit), the membrane was incubated with HRP-conjugated streptavidin per the manufacturer's instructions. Signal intensity was quantified by densitometry.

### Quantitation of IL-1α

Released IL-1α was measured using Human IL-1α ELISA Kit (Endogen) according to the manufacturer's instructions. Briefly, HMEEC medium was added into anti-human IL-1α precoated 96-well plates and incubated for one hour at room temperature. After adding biotinylated anti-cytokine antibody (provided in the kit), the 96-well plate was incubated with streptavidin-HRP solution for 30 minutes. Color was developed with TMB substrate solution after washing and the absorbance was measured at 450 nm minus 550 nm. The amount of IL-1α in unknown samples was determined after calculating the standard curve of reconstituted IL-1α diluents.

### Reverse transcription and real time quantitative PCR

Total RNA was extracted using the RNeasy kit (Qiagen, Valencia, Ca) and cDNA was synthesized using the TaqMan reverse transcription kit (Applied Biosystems, Foster City, CA). Taqman primers and probes for β-defensin 2 (DEFB4) (ABI assay number Hs00823638_m1 (NM_004942)), IL-1α (ABI assay number Hs00899844_m1 (NM_000575)) and cyclophilin D (ABI assay number Hs00234593_m1 (NM_005038)) were purchased from Applied Biosystems. Multiplex PCR was performed using 7500 Real Time PCR System (Applied Biosystems) and cycle threshold (CT) values were analyzed. CT values of DEFB4 or IL-1α were normalized with the internal control, and results were expressed as a fold-induction of mRNA quantity, taking the value of the non-treated group as 1.

### Promoter construct of DEFB4

Reporter construct of β-defensin 2 promoter (p2.7hBD2-luc) was generated as described [27]. Briefly, 5' flanking region (from -2625 to + 1) of β-defensin 2 (DEFB4) (AF_071216) was isolated by PCR amplification with the following primers: 5'-GAG GTA CCT CCA TCC TTT ACT GTG ATG ATG CC-3' (forward primer with Kpn1 tail), 5'-GAA AGC TTT GGC TGA TGG CTG GGA GCT TCA CCA (reverse primer with HindIII tail). The amplified product was subcloned into the multiple cloning site of the pGL3-Basic luciferase reporter plasmid (Promega, Madison, WI). The reporter construct was sequenced to verify number and orientation of inserted oligonucleotides.

### Transfection and Luciferase assay

All transient transfections were carried out in triplicate using TransIT-LT1 (Panvera, Madison, WI) according to the manufacturer's instructions. Cells were seeded into six-well plates at a density of 1.5 × 10^5 ^cell/well, and were transfected with p2.7hBD2-luc plasmid upon reaching 60% confluence. The transfected cells were not selected with antibiotics. NTHi lysate or IL-1α was added to the transfected cells 40 hrs post transfection. Cells were harvested after 8 hrs, and luciferase activity was measured using a luminometer (Pharmingen, La Jolla, CA) after mixing with luciferase substrate (Promega), per the manufacturer's instructions. The results were expressed as fold increase of luciferase activity, compared to non-treated controls.

### Blocking the effects of epithelial cell-derived IL-1α

Cell culture medium was collected 48 hrs after treatment with NTHi lysate. The medium was briefly centrifuged to remove the cellular debris and diluted 1:4. The diluted medium was then incubated with an anti-interleukin 1α antibody (20, 40 and 80 μg/ml) (Endogen, Woburn, MA) or with non-immunized rabbit IgG (20 μg/ml) (Sigma) for 30 min at room temperature. The antibody treated medium was then used to replace the medium on HMEEC transfected with the DEFB4 promoter construct. Cells were harvested after 8 hrs, and luciferase activity was measured as described above.

### Statistics

All experiments were carried out in triplicate. Results are expressed as mean ± standard deviation. Statistical analysis was performed using Student's t-test, with significance considered to be p < 0.01 or p < 0.05.

## Results

### Release of cytokines by NTHi lysate treatment in the middle ear epithelial cells and their effect on β-defensin 2 expression

**Figure 1:**
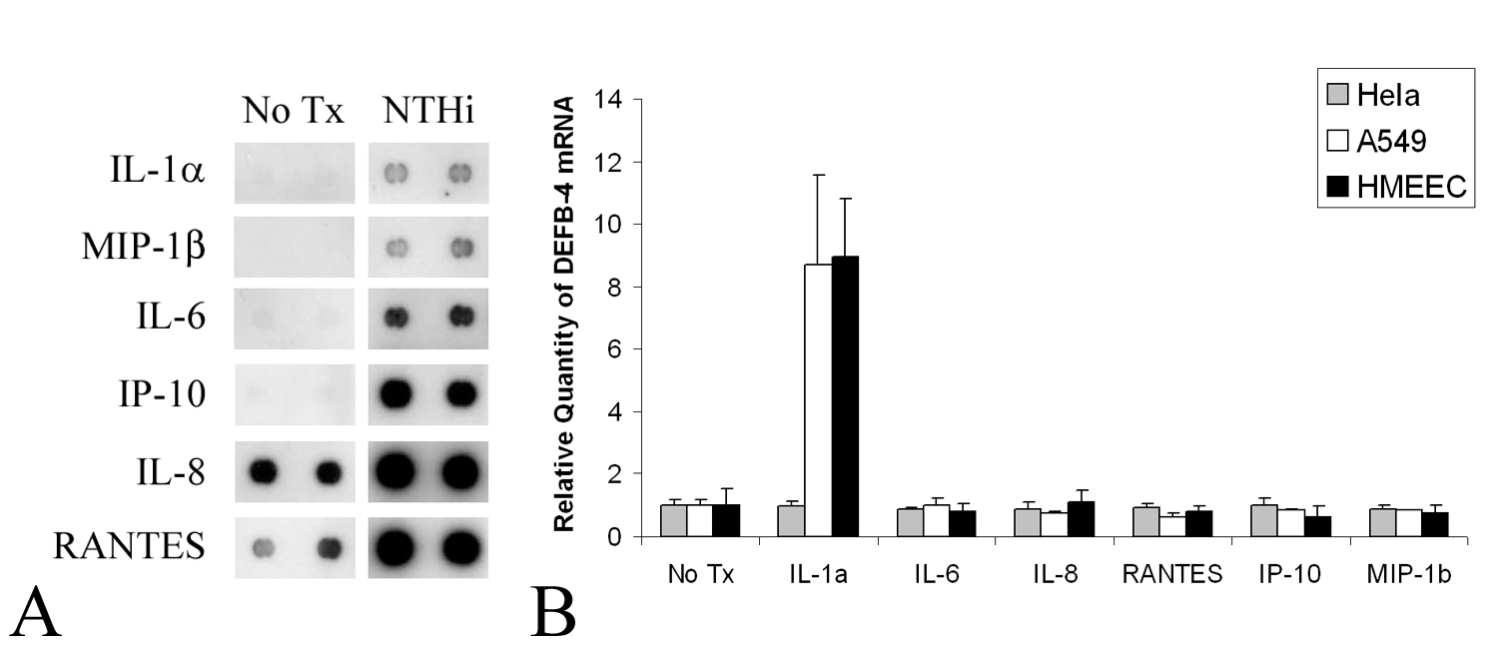
(A) Human cytokine macroarray analysis shows NTHi-induced release of cytokines by middle ear epithelial cells. Cell culture medium was harvested at 48 hrs after NTHi lysate treatment and the released cytokines were detected using asolid phase multiplexed protein assay in a sandwich ELISA format. Cells were treated with NTHi lysate (NTHi) or vehicle (No Tx). (B) Real time quantitative PCR analysis shows regulation of β-defensin 2 (DEFB4) mRNA by cytokines and chemokines. Only IL-1α treatment results in an up-regulation of β-defensin 2 (DEFB4) mRNA in HMEEC and A549 cells, but not in Hela cells.

Culture medium from human middle ear epithelial cells was collected 48 hours after NTHi lysate treatment and the release of cytokines was analyzed using a membrane blotted with various anti-cytokine and chemokine antibodies. IL-1α, MIP-1β, IL-6, IP-10, IL-8 and RANTES were present at significantly higher levels after stimulation with NTHi lysate (Figure 1A). Additionally, NTHi lysate treatment resulted in the secretion of IL-1α, but not IL-1β (data not shown). In order to test the effect of the cytokines on β-defensin 2 mRNA levels, human middle ear epithelial cells, Hela and A549 cells were treated with IL-1α, MIP-1β, IL-6, IP-10, IL-8 and RANTES. As shown in Figure 1B, only IL-1α treatment resulted in an up-regulation of β-defensin 2 (DEFB4) mRNA in HMEEC and A549 cells, but not in Hela cells.

### Upregulation of IL-1α by NTHi treatment in the middle ear epithelial cells

**Figure 2:**
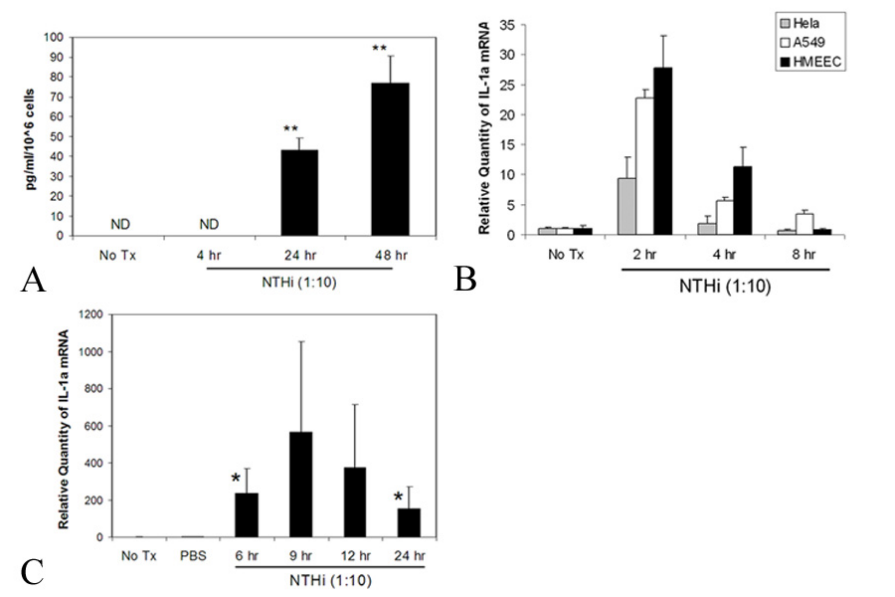
(A) HMEEC releases IL-1α by treatment with NTHi lysate. Cell culture medium was collected at 4, 24 and 48 hrs after treatment. Released IL-1α was quantified using ELISA (ND – not detectable). Treatment with NTHi lysate up-regulates IL-1α expression in a time dependent manner in Hela, A549 and HMEEC cells (B). Cells were treated with NTHi lysate for 2, 4 and 8 hours, and real time quantitative PCR was performed after RNA purification and reverse transcription. Inoculation of NTHi lysate into the middle ear results in the up-regulation of IL-1α mRNA in the mouse model (C). Mice were transtympanically inoculated with NTHi lysate and mucosal RNA was extracted after 6, 9, 12 and 24. Control animals received PBS. The RNA samples were reverse transcribed and analyzed by real time quantitative PCR. Values are given as mean ± standard deviation. N = 3. *: p < 0.05, **: p < 0.01.

Levels of secreted IL-1α were quantified by ELISA, and were at 46.0 ± 6.1 pg/ml/10^6 ^cells and 76.8 ± 13.9 pg/ml/10^6 ^cells (mean ± standard deviation) after 24 and 48 hours, respectively (Figure 2A). No IL-1 protein was detectable in media from the control cells or after 4 hours. To study the time course of the induction of IL-1α mRNA by NTHi components, Hela, A549 and HMEEC cells were treated with the bacterial lysate and RNA was extracted from the cells at different times. As shown in Figure 2B, treatment of the cells with NTHi lysate up-regulated IL-1α expression in a time dependent manner in Hela, A549 and HMEEC cells. To study the effect in animals, NTHi lysate was inoculated into the mouse middle ear. The middle ear mucosa was inflamed in all NTHi-treated mice, but effusion was not noted in all cases. As shown in Figure 2C, inoculation of NTHi lysate into the middle ear resulted in the up-regulation of IL-1α mRNA in the mouse model.

### Synergistic effect of IL-1α on NTHi-induced β-defensin 2 upregulation

**Figure 3:**
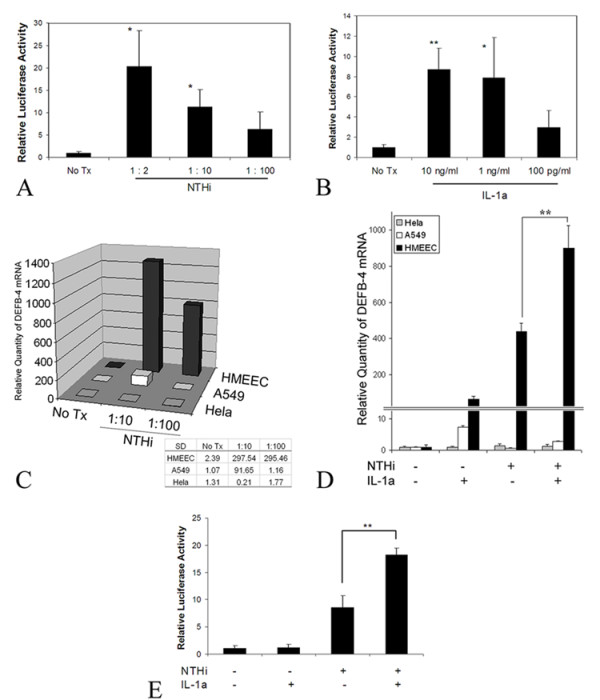
Individual and synergistic regulation of β-defensin 2 (DEFB4) by NTHi lysate and IL-1α. Luciferase assay of β-defensin 2 (DEFB4) promoter activity shows dose-dependent effect of NTHi lysate (A) and IL-1α(B) in HMEEC cells. Real time quantitative PCR (C) shows that HMEEC cells respond to NTHi treatment and up-regulate β-defensin 2 while Hela and A549 cells respond poorly. Insect: standard deviations (SD). Co-treatment of the cells with suboptimal doses IL-1α and NTHi lysate demonstrates a synergistic effect on the activation of the β-defensin 2 mRNA in HMEEC cells. The synergistic effect was not noted in Hela or A549 cells (D). Luciferase assay of HMEEC cells (E) shows synergistic transcriptional regulation by NTHi lysate (1:100 dilution) and IL-1α (50 pg/ml) co-treatment. Values are given as the mean ± standard deviation. N = 3. *: p < 0.05, **: p < 0.01.

**Figure 4:**
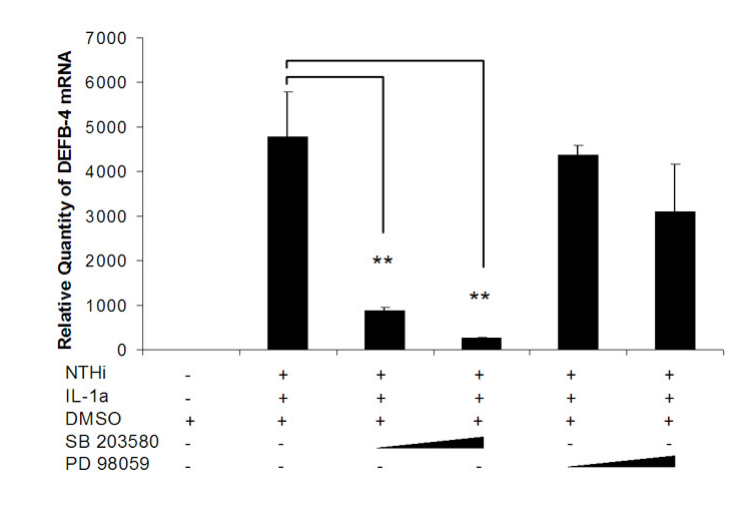
Regulation of the synergistic activity via the p38 MAP kinase pathway. Real time quantitative PCR shows that SB203580 (10 and 20 uM) blocks the synergistic effect of IL-1α and NTHi lysate, but PD98059 (10 and 20 uM) does not. Values are given as the mean ± standard deviation. N = 3. *: p < 0.05, **: p < 0.01. DMSO: dimethylsulfoxide.

To test if NTHi components and IL-1α could synergistically activate β-defensin 2 transcription, the human β-defensin 2 (DEFB4) promoter construct was transfected into HMEEC followed by treatment with NTHi lysate and IL-1α, separately or together. As shown in Figure 3, treatment of HMEEC with NTHi lysate (Figure 3A) or IL-1α (Figure 3B) separately resulted in an up-regulation of the β-defensin 2 promoter activity in a dose dependent manner. Interestingly, NTHi treatment of HMEEC resulted in the up-regulation of β-defensin 2 while Hela and A549 cells poorly responded (Figure 3C). Co-treatment of HMEEC cells with suboptimal doses IL-1α and NTHi lysate, however, had a synergistic effect on the activation of the β-defensin 2 mRNA (Figure 3D) and its promoter (Figure 3E). In contrast, this synergistic effect was not noted in Hela or A549 cells (Figure 3D). Moreover, the synergistic effect in HMEEC cells was inhibited by the p38 inhibitor (SB203580), but not by the MEK inhibitor (PD98059) (Figure 4). These results suggest that unlike IL-1α, which up-regulates β-defensin 2 transcription via the ERK pathway, the synergistic up-regulation of β-defensin 2 transcription by IL-1α and NTHi components is mediated via the p38 MAP kinase pathway.

### Inhibition of the effect of epithelial cell-derived β-defensin 2 inducing factor

**Figure 5:**
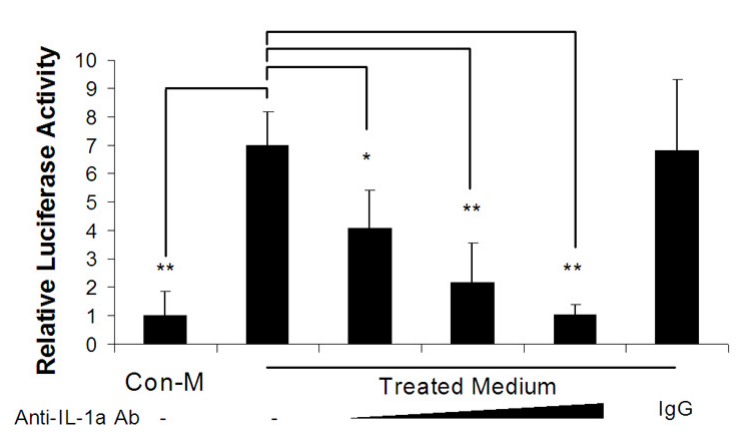
IL-1α is the protein secreted by NTHi-treated HMEEC cells that is responsible for the transcriptional activation of the β-defensin 2 (DEFB4) promoter. Treated medium – 1:4 dilution of the conditioned medium from cells treated with NTHi lysate for 48 hours. Control medium (Con-M) – 1:4 dilution of conditioned medium from non-treated cells. Anti-IL-1α antibody (20, 40 and 80 μg/ml), but not pre-immune IgG, can block the transcriptional activation of the β-defensin 2 promoter in a dose dependent manner. Values are given as the mean ± standard deviation. N = 3. *: p < 0.05, **: p < 0.01.

In order to prove that IL-1α was the cytokine responsible for the transcriptional activation of the β-defensin 2 promoter, HMEEC culture medium was collected 48 hrs after treatment of the cells with the NTHi lysate, diluted 1:4 with fresh media and treated with an IL-1α neutralizing antibody, or with pre-immune IgG. The antibody treated medium was next added to human middle ear epithelial cells transfected with the β-defesnisn 2 promoter construct. As shown in Figure 5, the activity of the β-defensin 2 (DEFB4) promoter was up-regulated by conditioned medium from NTHi-treated cells. Moreover, the observed induction could be blocked with anti IL-1α antibody, suggesting a major role for this cytokine in the up-regulation of β-defensin 2.

**Figure 6:**
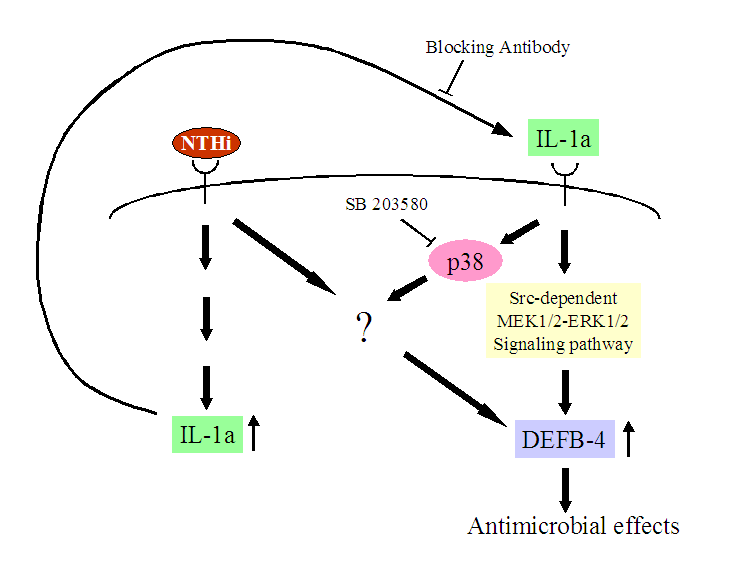
Schematic illustration showing pathways involved in the individual and synergistic effects of IL-1α and NTHi on β-defensin 2 (DEFB4) transcription in the middle ear epithelial cells.

Our results suggest that middle ear epithelial cells release IL-1α when stimulated by NTHi components, and that this cytokine acts in an autocrine/paracrine manner to synergistically up-regulate β-defensin 2 transcription. Furthermore, in contrast to IL-1α's effect on β-defensin 2 transcription, which is mediated via the ERK MAP kinase pathway, the synergistic effect of this cytokine on β-defensin 2 transcription by NTHi components appears to be mediated by the p38 MAP kinase pathway (Figure 6).

## Discussion

Our results suggest that middle ear epithelial cells release IL-1α when stimulated by NTHi components and that this cytokine acts in an autocrine/paracrine synergistic manner with NTHi to up-regulate β-defensin 2. Furthermore, in contrast to IL-1α that acts via the ERK MAP kinase pathway to induce β-defensin 2 transcription, the synergistic effect of this cytokine on β-defensin 2 up-regulation by NTHi molecules appears to be mediated by the p38 MAP kinase pathway (Figure 6). Such synergism may be a way of boosting the epithelial cells' initial response to NTHi and its components.

The signaling mechanisms appear to be more complicated in the presence of both exogenous and endogenous inducers. NTHi, an exogenous inducer and TNF-α, an endogenous inducer synergize with each other to induce NF-κB activation and consequently up-regulate inflammatory mediators including IL-1β and IL-8 as well as TNF-α [32]. NTHi first interacts with its receptors of host cells such as Toll-like receptor 2 (TLR2) [14], and leads to the activation of multiple signaling cascades resulting in the up-regulation of inflammatory cytokines such as TNF-α. The up-regulated TNF-α in response to NTHi synergize with NTHi to induce NF-κB via two distinct signaling pathways, the NIK-IKK-IκBα and the MEKK1-MKK3/6-p38 MAPK pathways [32].

Strains of NTHi, a small gram-negative bacterium, exist as commensal organisms in the human nasopharynx [33]. Nasopharyngeal colonization of NTHi may persist for prolonged periods of time, even in the antibody-containing mucosal secretions since the bacteria undergo antigenic variation. This can occur by several molecular mechanisms such as point mutation, gene amplification, phase variation or horizontal gene transfer and homologous recombination [34]. Although NTHi rarely causes life-threatening infections, it is nonetheless a clinically important pathogen since it causes otitis media in children and exacerbates chronic obstructive pulmonary disease in adults [35,36]. The interactions between NTHi and the host are not well understood. These interactions determine chronic colonization with or without inflammation or disease. The interaction of NTHi antigens and specific host molecules are likely to be involved in the transition of NTHi from a commensal to a pathogenic organism. Further studies are, however, needed to elucidate these mechanisms.

Our results showed that the induction of β-defenin 2 by NTHi is not a pan-epithelial response, in that Hela and A549 cells do not highly respond to NTHi lysates, whereas HMEEC cells do. In addition, although NTHi treatment up-regulated IL-1α expression in Hela, A549 and HMEEC cells, treatment of the cells with IL-1α resulted in the up-regulation of β-defensin 2 only in A549 and HMEEC, but not in Hela cells. These results indicate that expression patterns of receptors or signaling molecules is cell-specific and thus determines the responses to extracellular stimuli. Our results are in agreement with earlier reports of the hypo-responsiveness of A549 cells to lipopolysaccharide (LPS), and of A549 and normal human bronchial epithelial cells to Gram-positive group B Streptococci [37,38]. A deficiency in the expression of TLR2 and 4 may also explain the lack response of these cells to NTHi as well. Our experimental observation of the non-responsiveness of Hela cells to NTHi, is on the other hand different from previous observations demonstrating transcriptional up-regulation of β-defensin-2 by LPS [39] or activation of NF-κB by NTHi [14]. This apparent difference, however may be explained by Mineshiba and colleagues' use of LPS and the fact that Shuto and coworkers studied the nuclear translocation of NF-κB, which may not be in itself sufficient for the transcriptional activation of β-defensin-2 in Hela cells.

Although the autolysis of NTHi has not been documented, it is likely that NTHi undergoes autolysis similar to that seen with Haemophilus influenzae type b [40], S. pneumoniae [41] or E. coli [42]. Cytoplasmic, as well as membrane components of NTHi are thought to persist in the effusion and act as long lasting inducers of inflammation. This notion is supported by the fact that bacterial DNA is detectable in most sterile effusions [43], whereas endotoxin is detectable in 60% of the cases [44]. While the molecules (possibly surface antigens) of intact NTHi have been demonstrated to induce proinflammatory cytokines in human respiratory epithelial cells [45], other studies have shown that the cytoplasmic fraction of lysed NTHi also contains important molecules for stimulating epithelial cells [46,47]. The NTHi lysate used in this study was prepared by sonification of the bacteria and mimics normal pathophysiology.

Interleukin 1 is a central mediator of the inflammatory response and occurs in two forms. Although the acidic form, known as IL-1α, and the neutral form, IL-1β, only share 45% homology at the nucleic acid level, both proteins bind to the same receptor [48,49]. Furthermore, both proteins are pleiotropic and affect processes such as inflammation, immunity and hemopoiesis. Moreover, while IL-1β is active only in its secreted form, IL-1α is active as an intracellular precursor, a membrane-associated cytokine, as well as a secreted molecule.

Our results suggest a role for IL-1α but not IL-1β in the up-regulation of β-defensin 2 transcription. These results are consistent with the observations that IL-1α and not IL-1β, released from epithelial cells infected with respiratory syncytial virus, enhanced expression of intercellular adhesion molecule-1 in pulmonary epithelial cells [50]. Moreover, our data suggest that the induction of IL-1α production in middle ear epithelial cells and the autocrine/paracrine effect of this molecule may be one of the central events involved in the up-regulation of chemotactic factors for the recruitment of immune cells into the middle ear. It should be noted that although the concentration of IL-1α released into the culture medium was less than 100 pg/ml/10^6 ^cells, since the molecule is acting in an autocrine manner, its effective concentration is likely to be much higher locally.

In our previous studies, we demonstrated that IL-1α could up-regulate β-defensin 2 transcription via a Src-dependent MEK1/2-ERK1/2 signaling pathway [27]. Interestingly however, the present study shows that the p38 MAP kinase, not the ERK1/2 pathway is involved in synergism of IL-1α and NTHi components. Moreover, our preliminary results suggest that the p38 MAP kinase pathway is involved in the activation of β-defensin 2 transcription by NTHi (unpublished data). We postulate that the activation of ERK and p38 MAP kinase pathways can result in a synergistic up-regulation of β-defensin 2. These results point to the complexity of the signaling pathways that control the expression of innate immune molecules such as β-defensin 2 and indicate the need for further investigation into the regulation of β-defensin 2 by NTHi and IL-1α.

## Conclusion

We demonstrate that epithelial cell-derived IL-1α can synergistically act with NTHi components to up-regulate human β-defensin 2 (DEFB4) via the p38 MAP kinase pathway.

## List of abbreviations

OM: otitis media

NTHi: nontypeable *Haemophilus influenzae*

TLR: toll-like receptor

IL: interleukin

MAP kinase: mitogen-activated protein kinase

MEK: MAPK/ERK kinase

ERK: extracellular signal-regulated kinase

DEFB: human beta-defensin

IP: interferon-γ-inducible protein

RANTES: regulated upon activation, normally T-expressed, and presumably secreted

MIP: macrophage inflammatory protein

HMEEC: human middle ear epithelial cell line

IKK: I-kappa-B kinase

## Competing interests

The author(s) declare that they have no competing interests.

## Authors' contributions

SKM performed most of experiments and data analysis.

HYL prepared the lysate of NTHi, and assisted experiments related with bacteriology.

HP performed the cytokine macroarray analysis.

TT extracted RNA, and performed real time quantitative PCR.

RP performed transfection of the β-defensin 2 promoter construct and luciferase assay.

KC maintained the cell line and helped measuring IL-1α with ELISA.

AA oversaw study design and analysis, and drafted the manuscript.

DJL is recipient of NIDCD R01 DC005025-03, which supported this work and the studies were conducted in his laboratory, with him as the principal investigator.

## Pre-publication history

The pre-publication history for this paper can be accessed here:


